# π–π Stacking Distance and Phase Separation Controlled Efficiency in Stable All-Polymer Solar Cells

**DOI:** 10.3390/polym11101665

**Published:** 2019-10-12

**Authors:** Ke Zhou, Xiaobo Zhou, Xiaofeng Xu, Chiara Musumeci, Chuanfei Wang, Weidong Xu, Xiangyi Meng, Wei Ma, Olle Inganäs

**Affiliations:** 1 Biomolecular and Organic Electronics, IFM, Linköping University, SE-581 83 Linköping, Sweden; 2 State Key Laboratory for Mechanical Behavior of Materials, Xi’an Jiaotong University, Xi’an 710049, China; 3 Division of Surface Physics and Chemistry, IFM, Linköping University, SE-581 83 Linköping, Sweden; 4 Key Laboratory of Flexible Electronics (KLOFE) & Institute of Advanced Materials (IAM), Nanjing Tech University (NanjingTech), 30 South Puzhu Road, Nanjing 211800, China

**Keywords:** all-polymer solar cells, crystallinity, device stability, molecular packing structure, morphology, thermal annealing

## Abstract

The morphology of the active layer plays a crucial role in determining device performance and stability for organic solar cells. All-polymer solar cells (All-PSCs), showing robust and stable morphologies, have been proven to give better thermal stability than their fullerene counterparts. However, outstanding thermal stability is not always the case for polymer blends, and the limiting factors responsible for the poor thermal stability in some All-PSCs, and how to obtain higher efficiency without losing stability, still remain unclear. By studying the morphology of poly [2,3-bis (3-octyloxyphenyl) quinoxaline-5,8-diyl-alt-thiophene-2,5-diyl](TQ1)/poly[4,8-bis[5-(2-ethylhexyl)-2-thienyl]benzo[1,2-b:4,5-b′]dithiophene-alt-(4-(2-ethylhexyl)-3-fluorothieno[3,4-b]thiophene-)-2-carboxylate-2-6-diyl]] (PCE10)/PNDI-T10 blend systems, we found that the rearranged molecular packing structure and phase separation were mainly responsible for the poor thermal stability in devices containing PCE10. The TQ1/PNDI-T10 devices exhibited an improved PCE with a decreased π–π stacking distance after thermal annealing; PCE10/PNDI-T10 devices showed a better pristine PCE, however, thermal annealing induced the increased π–π stacking distance and thus inferior hole conductivity, leading to a decreased PCE. Thus, a maximum PCE could be achieved in a TQ1/PCE10/PNDI-T10 (1/1/1) ternary system after thermal annealing resulting from their favorable molecular interaction and the trade-off of molecular packing structure variations between TQ1 and PCE10. This indicates that a route to efficient and thermal stable All-PSCs can be achieved in a ternary blend by using material with excellent pristine efficiency, combined with another material showing improved efficiency under thermal annealing.

## 1. Introduction

Organic solar cells (OSCs) have attracted much attention over the last two decades due to their potential applications in solar energy conversion. Compared to other photovoltaic devices, OSCs can be easily fabricated using solution processing methods on flexible substrates, giving the advantages of light weight and large-area fabrication [1,2,3,4,5]. With the continuous efforts on materials synthesis, morphology control, and device structure optimization, the power conversion efficiency (PCE) of OSCs has reached over 15% very recently [6,7,8,9]. All-polymer solar cells (All-PSCs), which employ conjugated polymers as both donor and acceptor, are becoming one of the most important candidates with many advantages. Kim and other researchers found that, compared to their fullerene counterparts, All-PSCs usually exhibit the potential to achieve a high V_oc_ by tuning the molecular structure, strong light absorption, favorable mechanical properties, and simple large-area fabrication due to the favorable solution viscosity [10,11,12,13,14,15].

Holmes et al. [16] employed the polymer blend of poly(2-methoxy-5-(2’-ethyi)-hexyloxy-*p*-phenylenevinylene)(MEH-PPV)/cyano-substituted phenyl enevinylene (CN-PPV) as active layer to manufacture photovoltaic device in 1995, which was one of the pioneering works on All-PSCs. After that, the PCEs of All-PSCs have also continuously improved, from under 1% to over 10% during the last decade [17,18,19,20,21,22]. Recently, researchers found that all-polymer blend systems show an excellent thermal device stability, which is another obvious advantage for all-polymer solar cells [23]. Indeed, thermal annealing, as a commonly used post-treatment method, has been widely employed in OSCs to improve their PCEs, which is ascribed to the optimized blend morphology after thermal annealing. For example, Xia and Karuthedath et al. [24,25] found that thermal annealing could dramatically change morphology and improve device efficiency of TQ1/N2200 polymer blend, resulting from their superior charge separation and charge transport, as well as decreased charge recombination after thermal annealing. In particular, it will be easier to adjust the all-polymer blend morphology by using various thermal annealing temperatures if the glass transition temperature (T_g_) of each polymer is different [26]. Thermal annealing of fullerene-containing blend systems for a long time decreased the PCE due to the aggregation of fullerene molecules, while all-polymer blend systems showed a relatively stable morphology and thus excellent device stability even after a long period of thermal annealing. Kim et al. [27] have found that the PCE and thermal stability of poly[4,8-bis(5-(2-ethylhexyl)thiophen-2-yl)benzo[1,2-b:4,5-b′]dithiophene-alt-5-octyl4H-thieno[3,4-c]pyrrole-4,6(5H)-dione](PBDTTTPD)/(poly[[N,N′-bis(2-hexyldecyl)-naphthalene-1,4,5,8-bis(dicarboximide)-2,6-diyl]-alt-5,5′-thiophene] (P(NDI2HD-T)) all-polymer blend system could be further improved by adding an amount of fullerene less than 30 wt %, which resulted from the robust morphology, with well-dispersed fullerene in the amorphous portion of donor and acceptor polymers. Besides, Ma et al. [28] found that due to the excellent electron mobility in all-polymer system poly[(2,6-(4,8-bis(5-(2-ethylhexyl)thiophen-2-yl)benzo[1,2-b:4,5-b′]dithiophene))-alt-(5,5-(1′,3′-di-2-thienyl-5′,7′bis(2-ethylhexyl)benzo[1′,2′-c:4′,5′-c′]dithiophene-4,8-dione))] (PBDB-T)/ poly[[N,N′-bis(2-octyldodecyl)naphthalene1,4,5,8-bis(dicarboximide)-2,6-diyl]-alt-5,5′(2,2′-bithiophene)] (N2200), the device with high D:A blend ratio was also stable over 70 days under continuous heating at 80 °C in a glovebox without encapsulation, suggesting that all-polymer solar cells showed advantages in operational lifetime under thermal stress at various blend ratios. However, in some all-polymer blend systems decreased PCE induced by thermal annealing can, unfortunately, be observed; while the limiting factors responsible to the poor thermal stability still remain unclear, how to obtain higher efficiency without losing stability should be understood.

In this study, we employed two different polymer donors, poly[2,3-bis(3-octyloxyphenyl) quinoxaline-5,8-diyl-alt-thiophene-2,5-diyl] (TQ1) and poly[4,8-bis[5-(2-ethylhexyl)-2-thienyl]benzo[1,2-b:4,5-b′] dithiophene-alt -(4-(2-ethylhexyl)-3-fluorothieno[3,4-b]thiophene-)-2-carboxylate-2-6-diyl]] (PCE10, sometimes labeled PTB7-th), and the same naphthalene diimide-based polymer acceptor PNDI-T10 to investigate the effects of thermal annealing on the device performance, and identify the influencing factors of all-polymer device thermal stability. After careful characterization of the morphology and PCE in different donor/acceptor combinations, we found that the rearranged molecular packing structure, which is closely related to hole conductivity, and the phase separation were mainly responsible for the poor thermal device stability in PCE10-dominated blend systems. Besides, a maximum PCE could be achieved in a TQ1/PCE10/PNDI-T10 (1/1/1) ternary system after thermal annealing resulting from their favorable molecular interaction and the trade-off of molecular packing structure variations between TQ1 and PCE10.

## 2. Experimental Section

### 2.1. Device Preparation and Characterization

**Figure 1:**
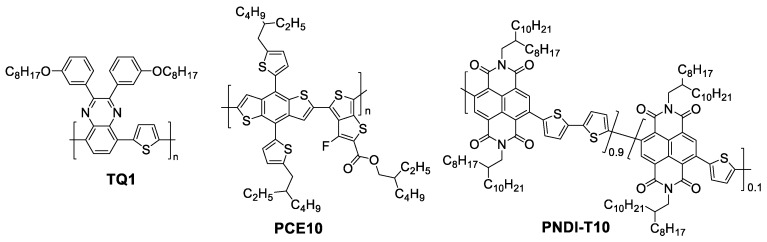
Chemical structures of poly[2,3-bis(3-octyloxyphenyl) quinoxaline-5,8-diyl-alt-thiophene-2,5-diyl] (TQ1), poly[4,8-bis[5-(2-ethylhexyl)-2-thienyl]benzo[1,2-b:4,5-b′] dithiophene-alt -(4-(2-ethylhexyl)-3-fluorothieno[3,4-b]thiophene-)-2-carboxylate-2-6-diyl]] (PCE10), and PNDI-T10.

Solar cells were fabricated on indium-tin oxide (ITO)-coated glass substrates, which were cleaned in advance by using detergent and then boiling in a mixture of water, ammonia (25%), and hydrogen peroxide (28%) with a blend ratio 5/1/1(*V*/*V*/*V*). Then, the poly(ethylene-dioxythiophene): poly (styrenesulfonate) (PEDOT: PSS, Baytron P4083) layer (35 nm) was spin-coated on the ITO glass and dried at 130 °C for 30 min. When the substrates cooled to room temperature, TQ1/PCE10/PNDI-T10 blend solutions with a various blend ratios (2/0/1, 1.5/0.5/1, 1/1/1, 0.5/1.5/1, and 0/2/1), which had a total concentration of 12 mg/mL in chloroform (CF), were then spin-cast on top of the PEDOT:PSS layer to produce a nearly 120 nm thick active layer, and thermal annealing was conducted at 120 °C for a range of times (10, 30, 120, 240, and 360 min) in a glovebox. Finally, a bilayer structure of LiF (0.6 nm)/Al (90 nm) was deposited atop the active layer by thermal evaporation in a vacuum under 10^−5^ Pa. The active area of each solar cell is 0.047 cm^2^. PCE10 was purchased from 1-materials company, TQ1 and PNDI-T10 were synthesized as described in previous reports [29,30]. The chemical structures of polymers are shown in Figure 1. The J–V curves were measured using a Keithley 2400 Source Meter under AM 1.5G illumination from a solar simulator (Model SS-50A, Photo Emission Tech. Inc. Moorpark, CA, USA) at 100 mW/cm^2^. EQE spectra were measured using a Newport Merlin lock-in amplifier.

### 2.2. Electroluminescence Measurement

The electroluminescence was recorded by a Newton electron-multiplying charge-coupled device (CCD) Si array detector cooled to −55 °C in conjunction with a Shamrock sr 303i spectrograph from Andor Technology served as the emission-detection system. A Keithley 2400 SourceMeter was employed to supply the biased voltage to the samples. The wavelength of this system was calibrated by using an argon lamp with a resolution better than 0.5 nm. Besides, the transmission of the entire fibre-monochromator-CDD system was further radiometrically calibrated by using an Optronic OL245M standard spectral irradiance lamp.

### 2.3. GIWAXS

The Grazing-incidence wide-angle X-ray scattering (GIWAXS) data of the thin film were obtained at beamline 7.3.3 at the advanced light source (ALS). Samples were prepared on Si substrates, which should be cleaned according to the same method with ITO-glass substrates, using the same blend solutions as those used in devices. In order to maximize the scattering intensity from the polymer blend samples, the 10 keV X-ray beam was incident at a grazing angle of 0.12–0.16°. The scattered x-rays were detected using a Dectris Pilatus 2M photon counting detector. The data were processed and analyzed using the NIKA software package.

### 2.4. Contact Angle Measurement

Water contact angle was characterized on a spin-coated film of each pure polymer by a CAM 200 optical contact angle meter using pure water. The surface energy of each polymer was evaluated from the contact angle, based on the previously reported method [31].

### 2.5. AFM

Atomic force microscopy (AFM) images were scanned using a Bruker INNOVA atomic force microscope using a tapping mode. Conductive-atomic force microscopy (C-AFM) characterization was performed in a Dimension 3100/Nanoscope IV system, equipped with a C-AFM module (current sensitivity 1 nA/V) (Bruker). Pt/Cr coated silicon probes with a spring constant of 0.2 N/m were used to measure current maps in contact mode by applying a constant load force of 2–5 nN. Current maps of the polymer blends were obtained by applying a constant bias to the ITO/PEDOT:PSS substrate while keeping the scanning AFM probe at ground. All the measurements were performed in dark in the ambient atmosphere.

## 3. Results and Discussions

### 3.1. Device Performance and Thermal Stability

**Figure 2:**
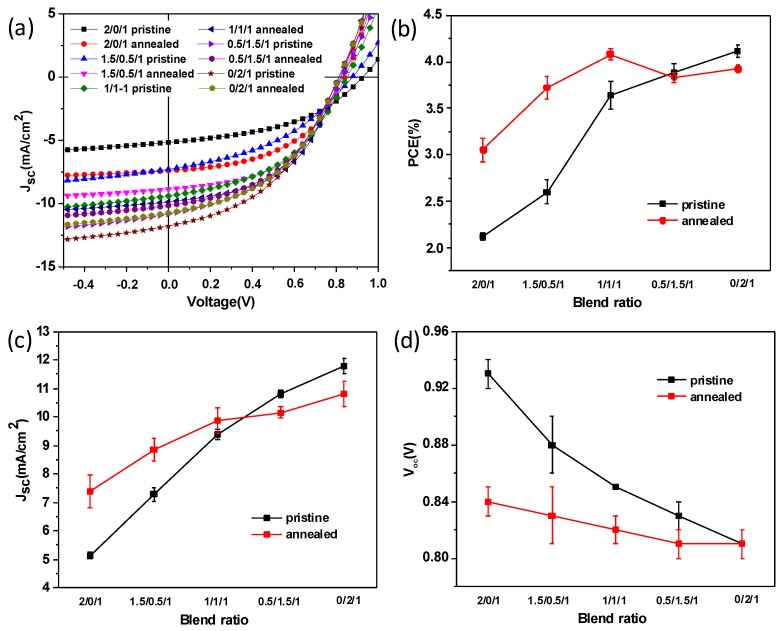
(**a**) *J*−*V* curves of devices with and without thermal annealing based on TQ1/PCE10/PNDI-T10 blend films with different blend ratios cast from CF and their variation trend of power conversion efficiency (PCE) (**b**), *J*_sc_ (**c**), and *V*_oc_ (**d**) with the change of blend ratio, the error bar was obtained based on 4 devices.

**Figure 3:**
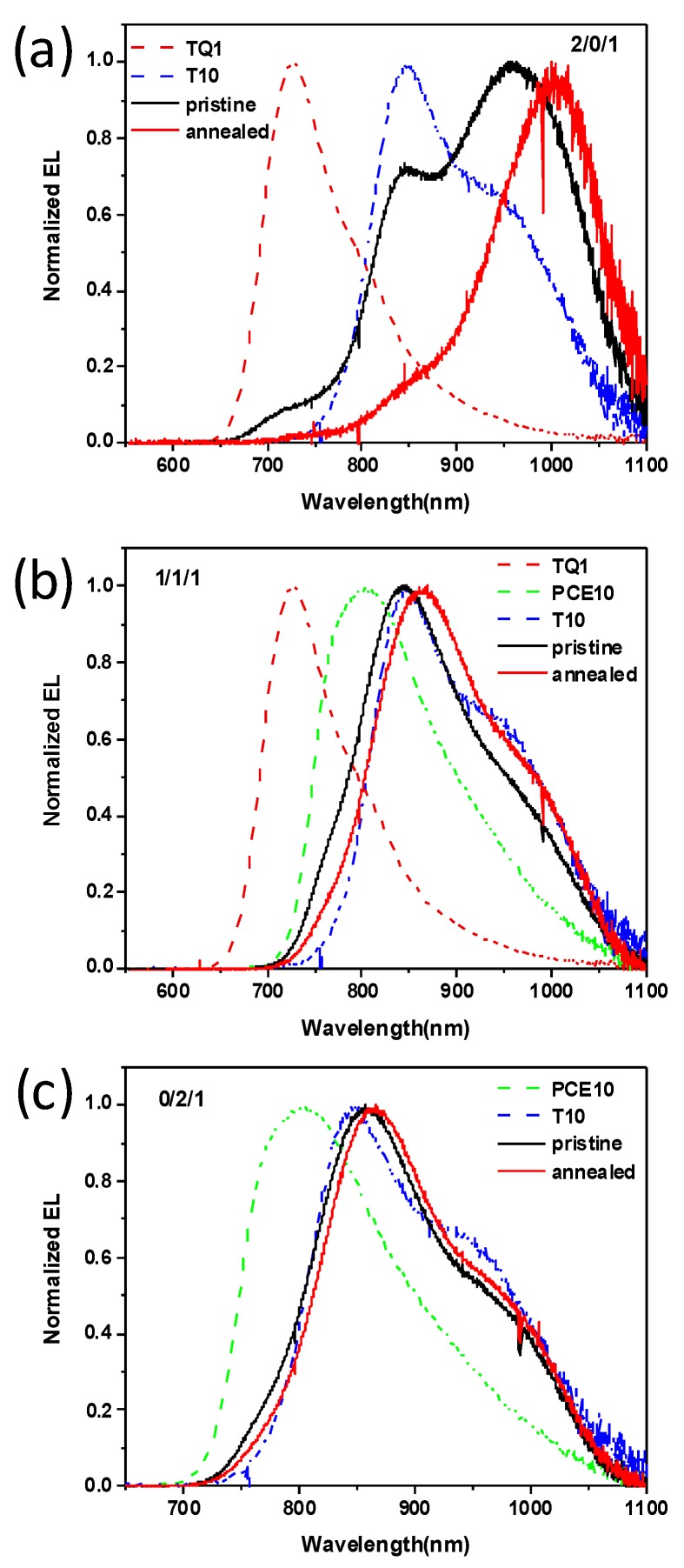
Electroluminescent (EL) spectra of TQ1/PCE10/PNDI-T10 blend films with different blend ratio: (**a**) 2/0/1, (**b**)1/1/1, and (**c**) 0/2/1.

In this study, we employed TQ1/PCE10 as donor and PNDI-T10 as acceptor to investigate the effect of thermal annealing on blend morphology and device stability, and tried to identify the influencing factor which was responsible for the device stability in terms of blend film morphology. As shown in Figure S1, the absorption spectrum of TQ1 film exhibits two peaks located at 360 and 621 nm, while the PNDI-T10 film shows red-shifted double absorption peaks located at 386 and 682 nm. This can be assigned to the charge-transfer transition at a low-energy band and the π→π * transition at a high-energy band. The PCE10 film shows a broad absorption peak ranging from 637 to 702 nm. First, we prepared devices based on TQ1/PCE10/PNDI-T10 combinations with different blend ratios, changing from 2/0/1, 1.5/0.5/1, 1/1/1, 0.5/1.5/1, to 0/2/1, *J*-*V* curves; the corresponding device parameters are shown in Figure 2 and Table 1. *V*_oc_ of the pristine and thermal annealed devices both decreased with the increasing content of PCE10. The *V*_oc_ difference between pristine and thermally annealed TQ1 devices was much larger than those of PCE10 devices. This can be ascribed to the easily changed energy level for TQ1 after thermal annealing, while the energy level of PCE10 is quite robust upon thermal annealing, which can be inferred from their peak position in electroluminescence spectra (see Figure 3) and their highest occupied molecular orbital (HOMO) level variations obtained with ultraviolet photoelectron spectroscopy (UPS) measurements (see Figure S2) [24]. Indeed, the morphology can also influence the device *V*_oc_ change. We found that for TQ1 binary blend films (AFM images, Figure 5), the morphology change is rather small upon thermal annealing. The root mean square (RMS) roughness values were calculated to be 0.568 and 0.560 nm for the pristine and annealed films, respectively. This suggests that thermal annealing has little effect on the phase separation in TQ1/PNDI-T10 blend films. Therefore, the *V*_oc_ variation in their devices upon thermal annealing was largely attributed to the shift of energy levels.

In addition, for the pristine devices, the PCE increased quasi-linearly from 2.12 to 4.18%, with the PCE10 content growing from 0 to 100%, while a maximum PCE of 4.08% could be found in the device with a blend ratio of 1/1/1 after thermal annealing. Interestingly, as shown in Figure 2b, the PCE of a device with a higher TQ1 content increased after thermal annealing, a similar increased PCE can also be found in all-polymer blend TQ1/N2200 after thermal annealing, as reported in previous work [24,25], in which the favorable charge separation and reduced geminate recombination after thermal annealing made a contribution. On the other hand, the PCE of devices with a higher PCE10 content decreased slightly, suggesting an inferior thermal stability for the PCE10-dominated devices. After carefully analyzing the specific device parameters, we found that FF of all the devices increased after thermal annealing; combined with the increased *J*_sc_, they both contributed an improved PCE in TQ1-dominated device after thermal annealing, while for the PCE10-dominated devices, the decreased *J*_sc_ was mainly responsible for the inferior PCE after thermal annealing. After comparing the present device efficiency with previous references, we found that TQ1-based binary devices had the nearly comparable efficiency with the reported data when using the same device geometry [24]. While the PCE10-based devices show relatively inferior efficiency, the main reasons can be ascribed to the materials with batch-to-batch variation and different D/A blend ratio [32]. However, the efficiency variations upon thermal annealing for TQ1/PCE10/PNDI-T10 blends are sufficient to track the underlying factors for the efficiency stability, which is related to the blend film morphology. We believe this will provide guidelines to achieve long-term device stability in terms of material design and processing condition optimization.

**Figure 4:**
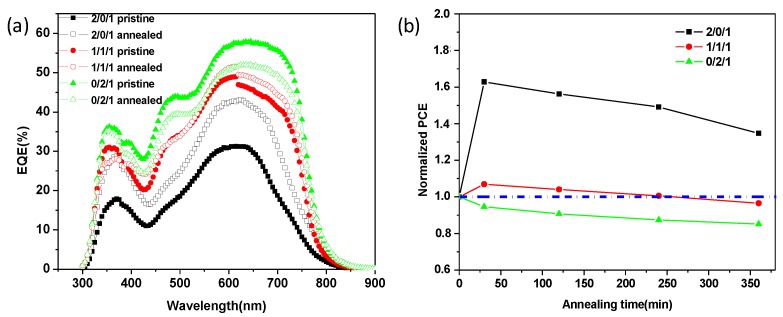
(**a**) EQE curves of devices with and without thermal annealing based on TQ1/PCE10/PNDI-T10 blend films with different blend ratios cast from CF; (**b**) Normalized PCE of devices with different thermal annealing time.

To further confirm the effects of thermal annealing on TQ1/PCE10/PNDI-T10 device performance and photocurrent, external quantum efficiencies (EQE) of devices without and with thermal annealing were measured (Figure 4a). TQ1/PNDI-T10 binary and TQ1/PCE10/PNDI-T10 (1/1/1) ternary devices showed an increased EQE from 31 to 43% and from 48 to 51%, which corresponded to the calculated *J*_sc_ from 5.37 to 7.82 mA/cm^2^ and from 9.52 to 10.14 mA/cm^2^, respectively. In contrast, the PCE10/PNDI-T10 binary device exhibited a decreased EQE from 57 to 50%, which corresponded to the calculated *J*_sc_ from 12.23 to 10.97 mA/cm^2^. The variations of *J*_sc_ values based on EQE calculation were consistent with those of *J*-*V* curves, indicating that thermal annealing really lead to a decreased *J*_sc_ and PCE in PCE10-dominated devices.

We also investigated the effect of different annealing time on the device stability for TQ1/PCE10/PNDI-T10 blend systems. As shown in Figure 4b, the values of normalized PCEs of TQ1/PNDI-T10 devices were all larger than 1 even after thermal annealing for 360 min, suggesting that TQ1/PNDI-T10 blend had an outstanding thermal stability. On the other hand, the values of normalized PCEs of PCE10/PNDI-T10 devices were all smaller than 1 even at a very short annealing time, indicating an inferior thermal stability compared to TQ1-dominated devices. For the ternary blend systems, the values of normalized PCEs were larger than 1 at a short annealing time, however, decreased <1 when the thermal annealing time was extended to 240 min, a probable trade-off between the superior thermal stability of TQ1 blends and poor thermal stability of PCE10 blends. Nevertheless, the PCE10-containing blend systems still showed a better thermal stability than their fullerene counterparts [33], which is the one of the most obvious advantages of polymer/polymer blend systems. Exploring and identifying why PCE10 blend systems have a relatively inferior thermal stability among other polymer/polymer blends will be of interest in the pursuit of further improvements in device thermal stability, in terms of material design and optimization of blend morphology.

### 3.2. Morphology and Molecular Packing Structure

Since the morphology of the active layer plays an important role in determining device performance and stability, characterization of the blend morphology and molecular packing information will be useful to understand the device thermal stability. As is well-known, solution processing is one of the clearest advantages of organic solar cells and has been widely used in various process methods, such as blade coating and spin coating. With the evaporation of solvent, donor and acceptor will undergo a phase separation, in which the blend morphology can be decided by the interaction parameters between donor and acceptor. Therefore, it’s useful to employ interaction parameters to predict the thin film morphology for a binary or ternary blend in the context of thermodynamics. Here, the contact angles (***θ***) of TQ1, PCE10, and PNDI-T10 neat films were measured, in order to evaluate their surface energy (***r***) and solubility parameter (*δ*) (Figure S3 and Table 2). TQ1 and PCE10 showed rather similar values of contact angles, which were 98.0° and 96.6°, respectively. The PNDI-T10 had a relatively larger value, namely 107.6°, than those of TQ1 and PCE10. Based on previous method [31,34] and the value of contact angle (see supporting information), we calculated the surface energy of each neat film, and the value was 23.5, 24.2, and 18.0 mN/m for TQ1, PCE10, and PNDI-T10, respectively. Furthermore, we obtained the solubility parameters of each neat film based on the value of their surface energy according to the exponential relationship between r and δ [35], the values of TQ1 and PCE10 were 17.8 and 18.0 M*Pa^1/2^, respectively, which had a quite small difference, suggesting that the interaction parameter between TQ1 and PCE10 molecules is relatively small. On the other hand, the solubility parameter of PNDI-T10 is much smaller than those of TQ1 and PCE10, which means that PNDI-T10 molecules will exhibit relatively large interaction parameters with TQ1 and PCE10 molecules. Therefore, with the evaporation of processing solvent, TQ1 and PCE10 molecules will tend to contact with each other rather than with PNDI-T10 molecules during phase separation in a ternary blend system, probably forming plenty of TQ1/PCE10 mixed domains. With the increasing content of PCE10 in the blend system, more PCE10 molecules will distribute in the TQ1/PCE10 mixed domains and contact with PNDI-T10 molecules, donor/acceptor interface in the bulk-heterojunction will probably change from TQ1/PNDI-T10 to PCE10/PNDI-T10, which is mainly responsible for the quasi-linear change of V_oc_, J_sc_, and PCE in the pristine devices as analyzed above. This trend is also similar to the previous results concerning the relationship between morphology and device parameter changes [36].

**Figure 5:**
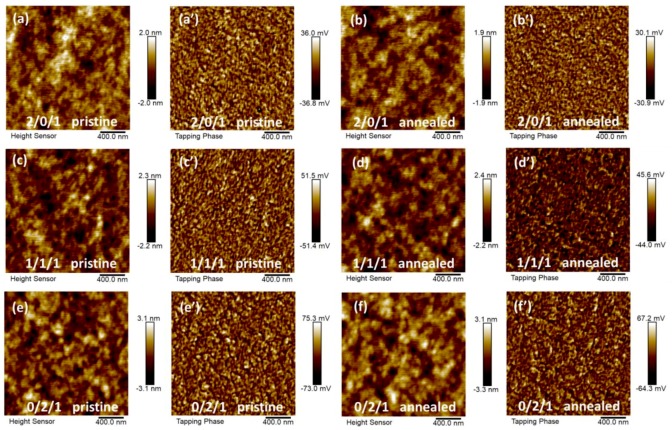
2 μm × 2 μm atomic force microscopy (AFM) height (**a**–**f**) and phase (**a’**–**f’**) images of TQ1/PCE10/PNDI-T10 blend films without and with thermal annealing for different blend ratio: (a,b) 2/0/1, (c,d)1/1/1, and (e,f) 0/2/1.

Atomic force microscopy (AFM) was employed to analyze the phase separation for different polymer blend films. As shown in Figure 5, all the blend films exhibit relatively flat surface morphology in the height images and small domain size in the phase images. The calculated root mean square (RMS) value enlarged gradually from 0.568, 0.622, to 0.891 for the pristine blend films with the increasing content of PCE10. In addition, from the phase images (Figure 5a’–f’) we found that the phase separation become more severe with the increasing content of PCE10. This indicates that the interaction parameter between PCE10 and PNDI-T10 is larger than that of between TQ1 and PNDI-T10. The RMS values obtained from 5 μm × 5 μm AFM height images also show the same trend. As shown in Figure S4, the RMS values are calculated to be 0.638/0.612, 0.671/0.716, and 1.02/1.10 nm for 2/0/1, 1/1/1, and 0/2/1 pristine/annealed films, respectively. More interestingly, the RMS values for 2/0/1 and 1/1/1 blend films exhibit smaller changes compared to those of 0/2/1 blend films upon thermal annealing. This further confirms that the trend of phase separation between TQ1 and PNDI-T10 is rather weak while strong phase separation can be found between PCE10 and PNDI-T10 molecules. Combined with the calculated solubility parameters (Table 2), in which the value of TQ1 is located between PCE10 and PNDI-T10, it can be inferred that TQ1 probably acts as a compatibilizer between PCE10 and the PNDI-T10 acceptor. This is one possible reason for the sTable 1/1/1 device performance upon thermal annealing.

**Figure 6:**
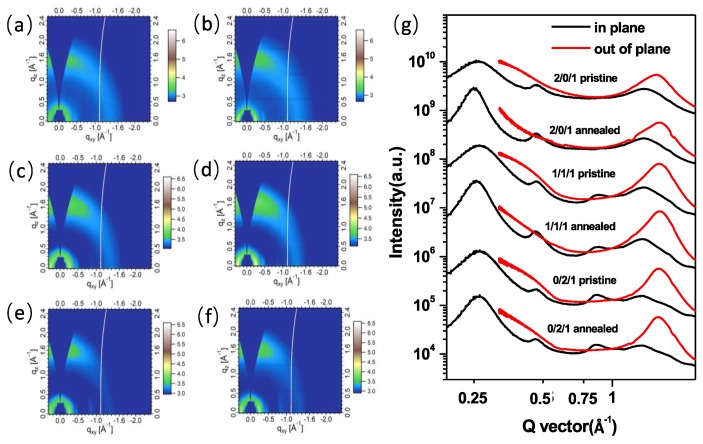
(**a**) GIWAXS 2D patterns of TQ1/PCE10/PNDI-T10 blend films without (**a**,**c**,**e**) and with (**b**,**d**,**f**) thermal annealing for different blend ratio: (**a**,**b**) 2/0/1, (**c**,**d**)1/1/1, and (**e**,**f**) 0/2/1; (**g**) scattering profiles of in-plane and out-of-plane of different blend films with and without thermal annealing.

It is well known that the molecular packing structure affects the device performance of all-polymer blend systems. Thus, we employed grazing incidence wide-angle X-ray scattering (GIWAXS) to study the molecular packing in TQ1/PCE10/PNDI-T10 blend films without and with thermal annealing, and obtained their coherence length and d-spacing in the in-plane and out-of-plane directions by fitting the GIWAXS data, the results are shown in Figure 6 and Figure S5, respectively. For the GIWAXS of neat films (see Figure S6), PCE10 and PNDI-T10 showed apparent peaks at 0.27 and 0.26 Å^−1^ within the in-plane direction, suggesting a (*100*) diffraction from the ordered lamellar packing, while another peak at 1.60 and 1.59 Å^−1^ within the out-of-plane direction can be found, which means a (*010*) diffraction from the π–π stacking. This indicates that both PCE10 and PNDI-T10 have a dominated face-on molecular orientation in the thin solid films. For the TQ1-based film, it showed a relatively weak in-plane (*100*) diffraction peak compared to those of PCE10 and PNDI-T10, while we can find a more pronounced (*010*) peak in out-of-plane direction than that of in-plane direction, which means TQ1 also have much more face-on molecular orientation. Since the peak positions for the neat films are quite similar, it’s hard to distinguish which material the peak comes from in the blend films, thus we used total diffraction peaks in the blend films to deduce the molecular packing information [36,37]. We noticed that all the blend films with and without thermal annealing exhibited very similar diffraction peaks at 0.25–0.26 Å^−1^ for the in-plane direction and 1.55–1.63 Å^−1^ for the out-of-plane direction (Figure 6), while after carefully fitting their peak position and full width at half maximum (FWHM), an interesting variation trend could be found for the coherence length and d-spacing. As is shown in Figure S5, coherence length and d-spacing of lamellar packing for binary and ternary blend all increased after thermal annealing, in which the improved amplitude in TQ1 blend was much more pronounced than that of the PCE10 blend. Besides, the coherence length of π–π stacking for binary and ternary blend increased as well after thermal annealing, however, the d-spacing of π–π stacking exhibited a different trend. For the TQ1 binary blend and TQ1/PCE10/PNDI-T10 ternary blend, d-spacing of π–π stacking both decreased, while in the PCE10 binary blend it increased slightly from 3.92 to 3.95 Å after thermal annealing, suggesting that the PCE10 blend featured a larger π–π ordered stacking distance after thermal annealing. It is therefore necessary to study the charge transport in devices with and without thermal annealing so as to illuminate the relationship between molecular packing structure and device thermal stability [38,39,40,41,42].

### 3.3. Hole Conductivity in Different Binary Blends

**Figure 7:**
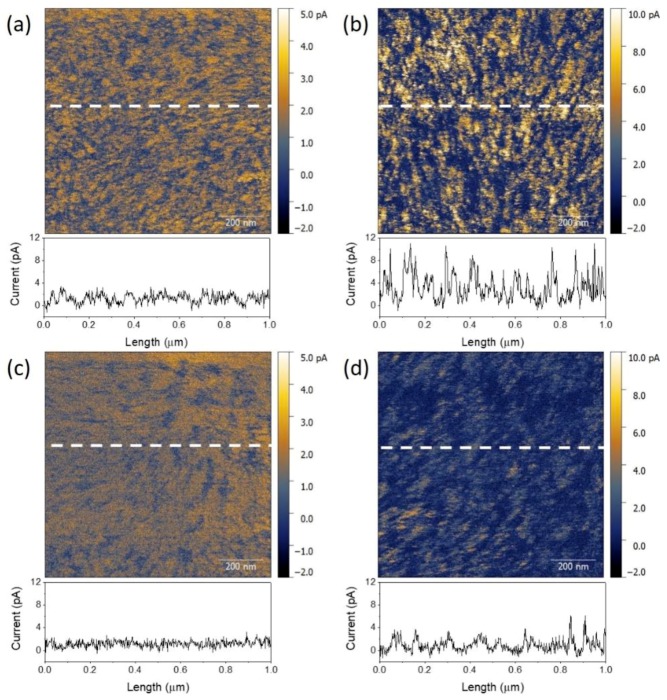
Current maps and their corresponding section profiles (below) of TQ1/PNDI-T10 = 2/1 (**a**,**c**) and PCE10/PNDI-T10 = 2/1(**b**,**d**) blend films without (**a**,**b**) and with (**c**,**d**) thermal annealing.

We first used space-charge-limited current (SCLC) to study the charge transport in different polymer blends (see Figure S7). However, we could not find the region with a slope of 2 in the trap-limited area for the hole-only devices. Therefore, conductive atomic force microscopy (C-AFM) was used to study the charge conductivity in TQ1/PNDI-T10 and PCE10/PNDI-T10 blend films. The current map for the blend films can provide the morphology and charge conductivity information in the vertical direction with respect to the film plane. In these maps, higher current regions (brighter area) represent donor-rich phase and lower current regions (darker area) represent acceptor-rich phase, because of the favored injection and extraction of holes enabled by the ITO/PEDOT: PSS and Pt/Cr electrodes. As shown in Figure 7, pristine TQ1/PNDI-T10 and PCE10/PNDI-T10 blend films both showed current maps where nanometer-sized domains of higher and lower conductivity are evenly distributed. The current spatial variations, described by the root mean square (RMS), were 0.95 and 2.50 pA for the two samples respectively. After annealing, both films exhibited a lower contrast between the domains and their corresponding RMS values reduced to 0.76 and 1.24 pA for TQ1/PNDI-T10 and PCE10/PNDI-T10 blend films. The corresponding section profiles along the line marked in the C-AFM images also featured a decreased noise after thermal annealing, suggesting a more uniform surface morphology. On the other hand, the average current of pristine TQ1/PNDI-T10 blend film, with a value of 1.19 pA, was smaller than that of the pristine PCE10/PNDI-T10 blend film (2.75 pA), indicating a more favorable hole conductivity in the latter one. However, after thermal annealing the average current of PCE10/PNDI-T10 blend film decreased to 1.05 pA, while the TQ1/PNDI-T10 blend film kept a similar value (1.17 pA), which can be ascribed to the inferior hole conductivity for the PCE10-rich phase after thermal annealing. This indicates that π–π stacking distance really affects the charge conductivity in blend films. In a decreased π-π stacking distance, the holes will hop and transport within this more ordered packing more easily and fast than those of increased packing distance, which explains the decreased hole conductivity for the PCE10/PNDI-T10 blend after annealing and consequently the inferior PCE for the PCE10-dominated devices.

In addition, charge recombination in TQ1/PNDI-T10 and PCE10/PNDI-T10 blend film was also studied by measuring the light intensity dependence of *J*_sc_ for the pristine and thermally annealed devices, with results shown in Figure S8. For the *J*_sc_, both of them showed a power law dependence on the light intensity, and the fitted exponents α for pristine and annealed PCE10/PNDI-T10 devices are 0.96 and 0.95, respectively, while for the TQ1/PNDI-T10 devices with and without annealing, it showed the same α value of 0.94. This indicates that the bimolecular recombination was very similar in TQ1/PNDI-T10 and PCE10/PNDI-T10 device with and without thermal annealing. Besides, the light-dependent *V*_oc_ for TQ1/PNDI-T10 and PCE10/PNDI-T10 device was very similar and showed a closed value of slope (~1.00 *kT*) for the pristine and annealed devices, suggesting that both of the binary blend system featured a dominant bimolecular recombination.

## 4. Conclusions

In this study, we investigated the variations of morphology and device performance with thermal annealing for TQ1/PCE10/PNDI-T10 blend system, and found that the rearranged molecular packing structure and phase separation were mainly responsible to the poor thermal device stability in PCE10-containing devices. The TQ1/PNDI-T10 devices exhibited improved PCE with the decreased π–π stacking distance from 4.06 to 3.94 Å after thermal annealing, and PCE10/PNDI-T10 devices showed a better pristine PCE. hHowever, thermal annealing induced an increased π–π stacking distance and thus inferior hole conductivity, and lower PCE could be observed. Thus, a maximum PCE could be obtained in a TQ1/PCE10/PNDI-T10 (1/1/1) ternary system after thermal annealing resulting from their favorable molecular interaction and the trade-off of molecular packing structure variations between TQ1 and PCE10. This indicates that a better efficient and thermal stable All-PSC can be achieved in a ternary blend by using material with an excellent pristine efficiency combined with another one showing improved efficiency under thermal annealing.

## Figures and Tables

**Table 1:** Device parameters of TQ1/PCE10/PNDI-T10 blend films with different blend ratios cast from chloroform (CF). The average values are obtained based on 4 devices.

		V_oc_(V)	J_sc_ (mA/cm^2^)	FF	PCE/Average (%)
2/0/1	pristine	0.93	5.13	0.45	2.12/2.10
	annealed	0.84	7.39	0.49	3.05/3.00
1.5/0.5/1	pristine	0.88	7.28	0.41	2.60/2.59
	annealed	0.83	8.86	0.51	3.72/3.68
1/1/1	pristine	0.85	9.39	0.46	3.64/3.57
	annealed	0.82	9.86	0.50	4.08/4.07
0.5/1.5/1	pristine	0.82	10.97	0.44	3.97/3.90
	annealed	0.81	10.38	0.46	3.88/3.86
0/2/1	pristine	0.81	12.05	0.43	4.18/4.13
	annealed	0.81	10.82	0.45	3.96/3.93

**Table 2:** Contact angle, surface energy, and solubility parameters of TQ1, PCE10, and PNDI-T10 pure film.

	θ ^°^	r (mN/m)	δ (M Pa^1/2^)
TQ1	98.0	23.5	17.8
PCE10	96.6	24.2	18.0
PNDI-T10	107.6	18.0	15.6

## Supplementary Materials

The following are available online at https://www.mdpi.com/2073-4360/11/10/1665/s1, Figure S1: Absorption spectra of TQ1, PCE10, and PNDI-T10 neat films. Figure S2: Low and high energy parts of UPS spectra for neat TQ1 and PCE10 films under -10 V bias. Figure S3: Contact angle of TQ1, PCE10, and PNDI-T10 neat films. Figure S4: 5μm×5μm AFM height and phase images of TQ1/PCE10/PNDI-T10 blend films without and with thermal annealing for different blend ratio. Figure S5: Coherence length and d-spacing of (100) and (010) peaks corresponding to 1D profiles in fig. 5g. Figure S6: GIWAXS of TQ1, PCE10, and PNDI-T10 neat films. Figure S7: hole-only devices and electron-only devices for the all-polymer blends with and without thermal annealing. Figure S8: Light intensity dependence of J_sc_ and V_oc_ based on TQ1/PNDI-T10=2/1 and PCE10/PNDI-T10=2/1 devices.
