# Supplementary material for: π–π Stacking Distance and Phase Separation Controlled Efficiency in Stable All-Polymer Solar Cells
Source: Polymers (Basel). 2019 Oct 12;11(10):1665. doi: 10.3390/polym11101665 (PMC6835461; doi:10.3390/polym11101665)
Supplement: Supplementary file 1 [file polymers-11-01665-s001.zip › polymers-578000-SI.pdf]

Supporting Information

π–π Stacking Distance and Phase Separation Controlled Efficiency in Stable All-Polymer Solar Cells

Ke Zhou [1,2,*], Xiaobo Zhou [2], Xiaofeng Xu [1], Chiara Musumeci [1], Chuanfei Wang [3], Weidong Xu [1,4], Xiangyi Meng [2], Wei Ma [2,*] and Olle Inganäs [1,*]

[1] Biomolecular and Organic Electronics, IFM, Linköping University, SE-581 83 Linköping, Sweden; xuxiaofeng@ouc.edu.cn (X.X.); chiara.musumeci@liu.se (C.M.); wiedong.xu@liu.se (W.X.)

[2] State Key Laboratory for Mechanical Behavior of Materials, Xi'an Jiaotong University, Xi'an 710049, China; ab1034525827@stu.xjtu.edu.cn (X.Z.); xiangyi3216@163.com (X.M.)

[3] Division of Surface Physics and Chemistry, IFM, Linköping University, SE-581 83 Linköping, Sweden; chuanfei.wang@liu.se

[4] Key Laboratory of Flexible Electronics (KLOFE) & Institute of Advanced Materials (IAM), Nanjing Tech University (NanjingTech), 30 South Puzhu Road, Nanjing 211800, China

* Correspondence: msekzhou@mail.xjtu.edu.cn (K.Z.); msewma@xjtu.edu.cn (W.M.); olle.inganas@liu.se (O.I.)

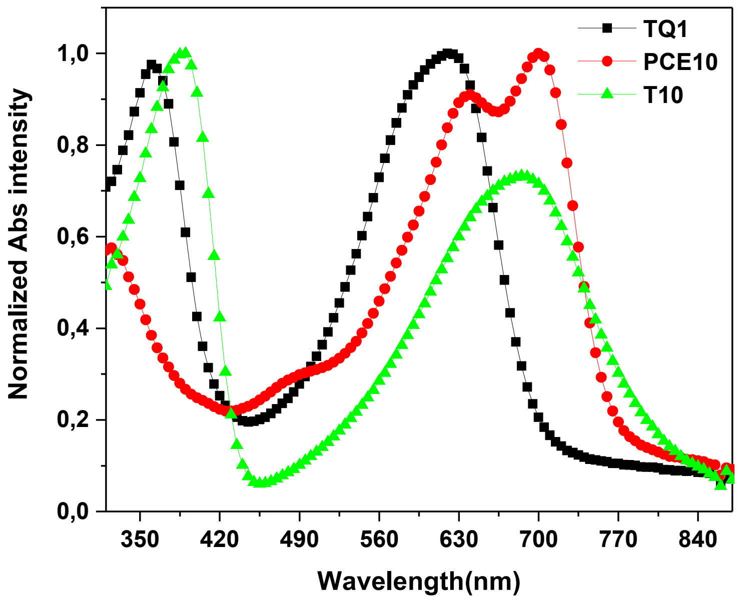

Figure S1 Absorption spectra of TQ1, PCE10, and PNDI-T10 neat films.

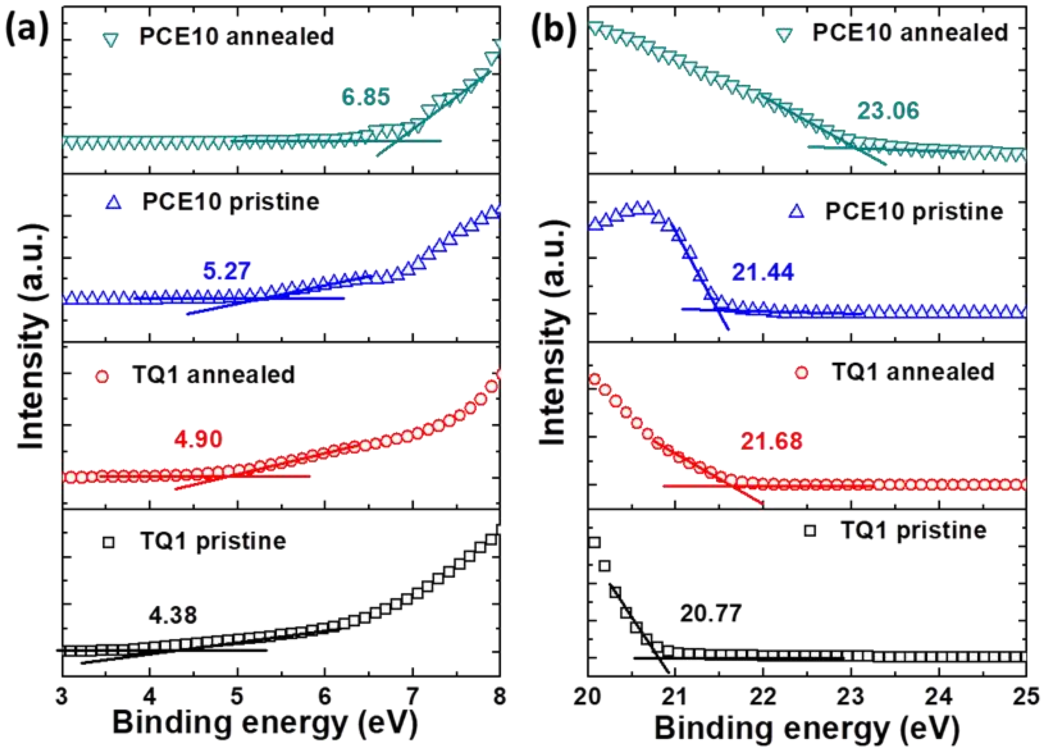

Figure S2 Low (a) and high (b) energy parts of UPS spectra for neat TQ1 and PCE10 films under -10 V bias. The HOMO energy levels are calculated based on the equation: $E_{HOMO} = hv - (E_{cutoff} - E_{onset})$, in which E_{cutoff} and E_{onset} can be found in the high and low energy parts of UPS spectra, respectively. The calculated values of HOMO energy are 4.81 and 4.54 eV for TQ1 without and with thermal annealing, respectively, suggesting that thermal annealing induced an up-shifted HOMO level. For PCE10 films, the values of HOMO energy are 5.03 and 4.99 eV for the pristine and annealed films, respectively, which indicates that the energy level of PCE10 is rather robust upon thermal annealing compared to its TQ1 counterparts.

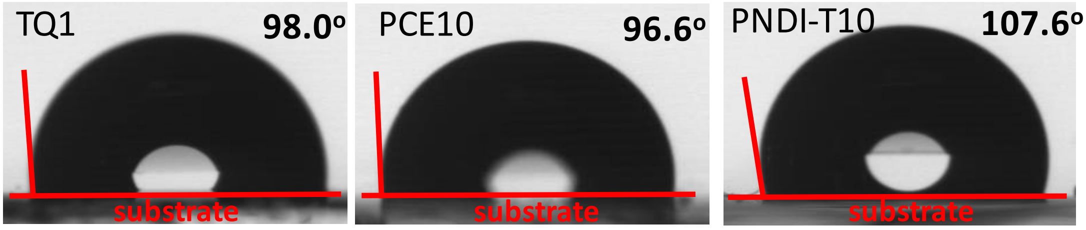

Figure S3 Contact angle of TQ1, PCE10, and PNDI-T10 neat films.

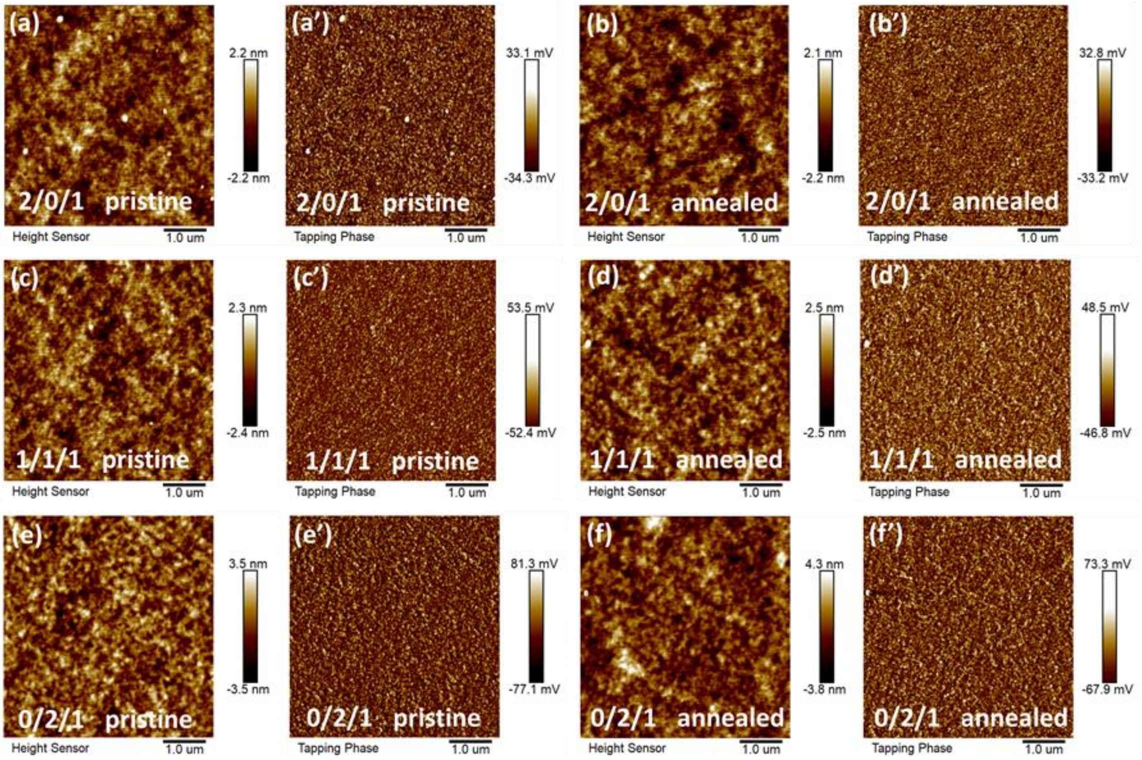

Figure S4. 5µm×5µm AFM height (a-f) and phase (a'-f') images of TQ1/PCE10/PNDI-T10 blend films without and with thermal annealing for different blend ratio: (a-b) 2/0/1, (c-d) 1/1/1, and (e-f) 0/2/1. The root-mean-square roughness (RMS) values are calculated to be 0.638/0.612, 0.671/0.716, and 1.02/1.10 nm for 2/0/1, 1/1/1, and 0/2/1 pristine/annealed films, respectively.

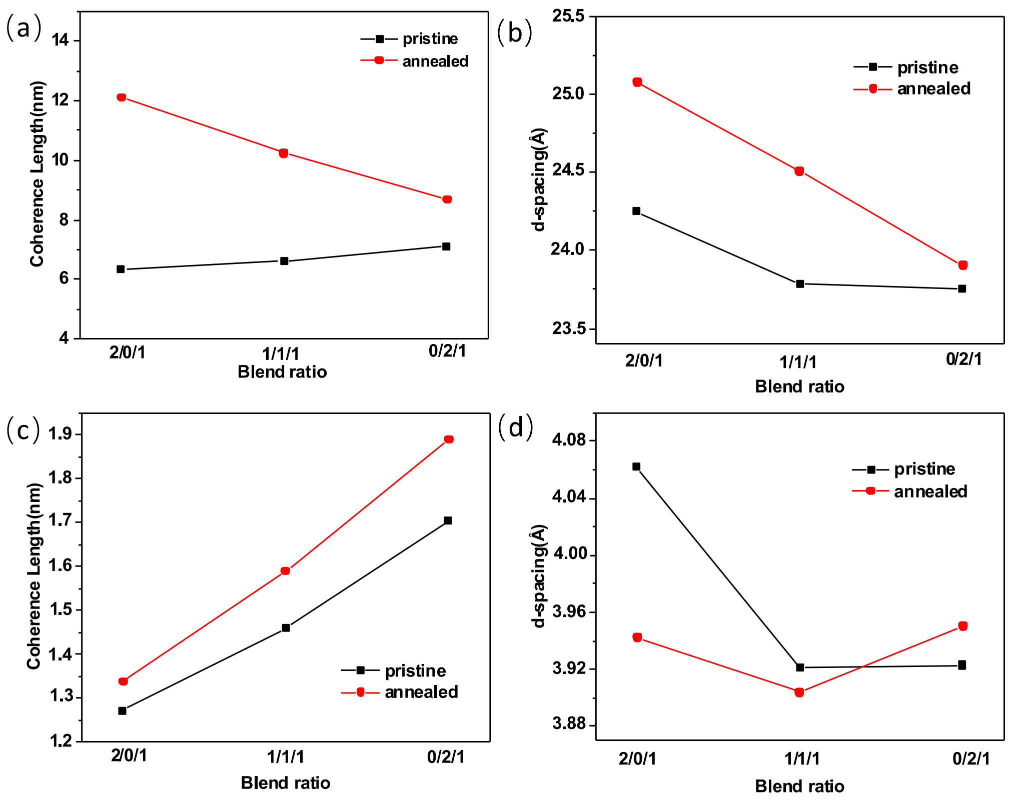

Figure S5 Coherence length and d-spacing of (*100*) (a-b) and (*010*) (c-d) peaks corresponding to 1D profiles in fig. 5g.

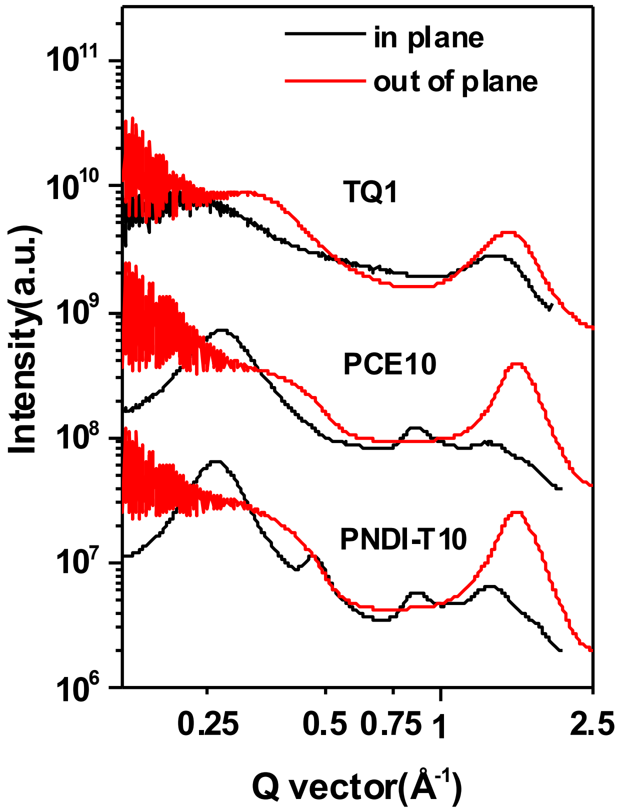

Figure S6 GIWAXS of TQ1, PCE10, and PNDI-T10 neat films.

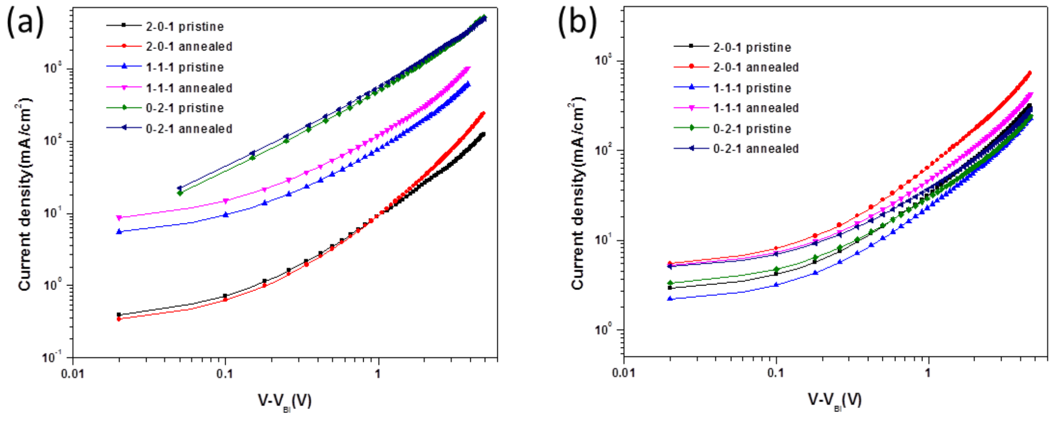

Figure S7 (a) hole-only devices and (b) electron-only devices for the all-polymer blends with and without thermal annealing.

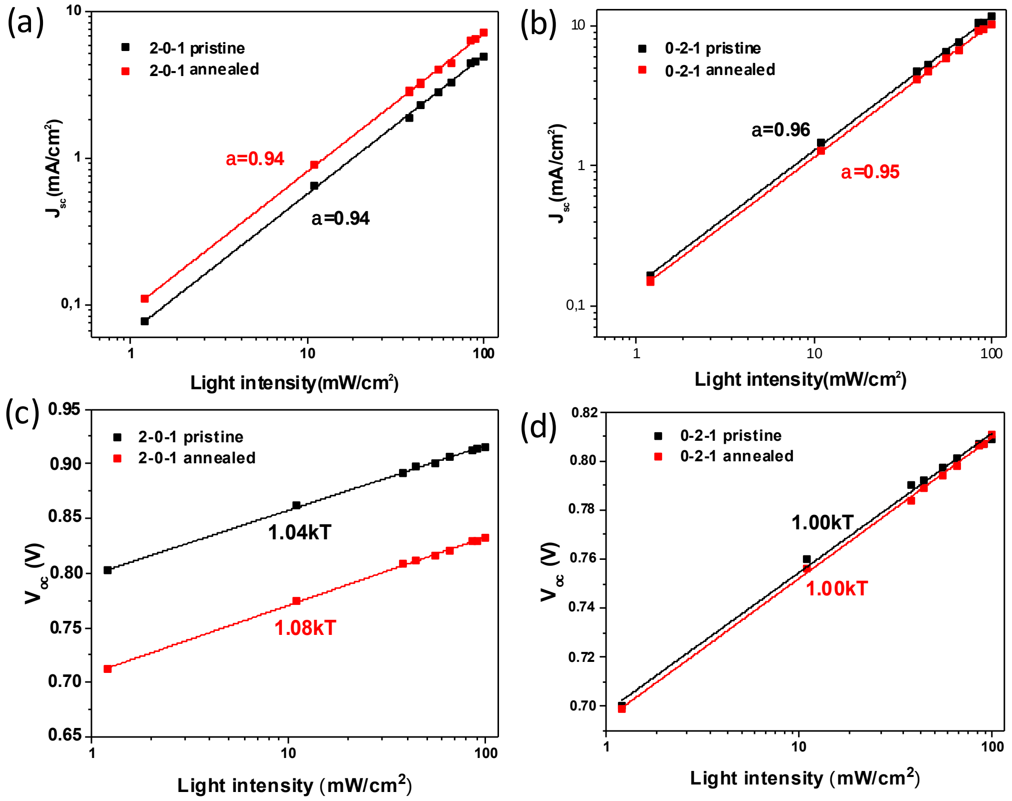

Figure S8 Light intensity dependence of J_{sc} and V_{oc} based on TQ1/PNDI-T10=2/1 (a, c) and PCE10/PNDI-T10=2/1 (b, d) devices.

References:

1. Zheng, L.; Zhu, T.; Xu, W.; Liu, L.; Zheng, J.; Xiong Gong, X.; Wudl, F. Solution-Processed Broadband Polymer Photodetectors with a Spectral Response of up to 2.5µm by a Low Bandgap Donor–Acceptor Conjugated Copolymer. *J. Mater. Chem. C*, 2**018**, *6*, 3634-3641.

2. Xu, W.; Yan, C.; Kan, Z.; Wang, Y.; Lai, W. Y.; Huang, W., High Efficiency Inverted Organic Solar Cells with a Neutral Fulleropyrrolidine Electron-Collecting Interlayer. *ACS Appl. Mater. Interfaces*, **2016**, *8*, 14293-14300.

3. Proctor, C. M.; Kuik, M.; Nguyen, T. C., Charge carrier recombination in organic solar cells. *Progress in Polymer Science*, **2013**, *38*, 1941-1960.
